# Determinants of clinical practice guidelines’ utilization for the management of musculoskeletal disorders: a scoping review

**DOI:** 10.1186/s12891-021-04204-w

**Published:** 2021-06-01

**Authors:** Delphine Sorondo, Cyrille Delpierre, Pierre Côté, Louis-Rachid Salmi, Christine Cedraschi, Anne Taylor-Vaisey, Nadège Lemeunier

**Affiliations:** 1 UMR1295, Toulouse III University, Inserm, Equipe EQUITY, Equipe constitutive du CERPOP, Toulouse, France; 2 Institut Franco-Européen de Chiropraxie, 72 chemin de la Flambère-31,300, Toulouse, France; 3 Faculty of Health Sciences, Ontario Tech University, Oshawa, Ontario Canada; 4 Centre for Disability Prevention and Rehabilitation at Ontario Tech University and the Canadian Memorial Chiropractic College, Oshawa and Toronto, Ontario Canada; 5 Univ. Bordeaux, ISPED, Centre INSERM U1219-Bordeaux Population Health, F-33000 Bordeaux, France; 6 CHU de Bordeaux, Pôle de santé publique, Service d’Information Médicale, F-33000 Bordeaux, France; 7 Division of General Medical Rehabilitation, University of Geneva, Geneva, Switzerland; 8 Division of Clinical Pharmacology and Toxicology, Multidisciplinary Pain Centre, Geneva University Hospitals, Geneva, Switzerland

**Keywords:** Clinical practice guidelines - musculoskeletal disorders, Adherence

## Abstract

**Context:**

Many clinical practice guidelines have been developed for the management of musculoskeletal disorders (MSDs). However, there is a gap between evidence-based knowledge and clinical practice, and reasons are poorly understood. Understanding why healthcare providers use clinical practice guidelines is essential to improve their implementation, dissemination, and adherence.

**Aim:**

To identify determinants of clinical practice guidelines’ utilization by health care providers involved in the assessment and management of MSDs.

**Method:**

A scoping review of the literature was conducted. Three databases were searched from inception to March 2021. Article identification, study design, methodological quality, type of healthcare providers, MSDs, barriers and facilitators associated with guidelines’ utilization were extracted from selected articles.

RESULTS: 8671 citations were retrieved, and 43 articles were selected. 51% of studies were from Europe, and most were quantitative studies (64%) following a cross-sectional design (88%). Almost 80% of articles dealt with low back pain guidelines, and the most studied healthcare providers were general practitioners or physiotherapists. Five main barriers to guideline utilization were expressed by providers: 1) disagreement between recommendations and patient expectations; 2) guidelines not specific to individual patients; 3) unfamiliarity with “non-specific” term, or with the bio psychosocial model of MSDs; 4) time consuming; and 5) heterogeneity in guideline methods. Four main facilitators to guideline utilization were cited: 1) clinician’s interest in evidence-based practice; 2) perception from clinicians that the guideline will improve triage, diagnosis and management; 3) time efficiency; and 4) standardized language.

**Conclusion:**

Identifying modifiable determinants is the first step in developing implementation strategies to improve guideline utilization in clinical practice.

**Supplementary Information:**

The online version contains supplementary material available at 10.1186/s12891-021-04204-w.

## Contributions to the literature’ statement

- *lack of studies focusing on both barriers and facilitators of all practitioners involved in the management of one pathology;*
- *No study has investigated the barriers and facilitators of guidelines’ utilization in all healthcare practitioners managing MSDs;*
- *Here, the emphasis is putted on the management and the assessment of a disorder not depending on a profession;*
- *Providing an overview of determinants related to guideline utilization by health care providers;*
- *Odd situation: some determinants are both barriers and facilitators.*

## Introduction

Musculoskeletal disorders (MSDs) are the leading cause of years lived with disability in the world [1]. These disorders affect people of all ages but the prevalence peaks in older individuals [2]. The Centers for Disease Control and Prevention (CDC) define MSDs as grade I-II sprain/strains, tendinitis, tendinosis, tendinopathy, neuropathies and nonspecific pain of the upper extremity, lower extremity, or spine [3]. MSDs can impact health-related quality of life, social interactions and work habilitations [2]. Consequently, MSDs have economic repercussions for patients and society [2].

Many clinical practice guidelines exist to inform the management of MSDs [4–9]. Guidelines are defined as guidance documents for clinical practice [10]. They are generally developed by synthesizing the best evidence on patient-centered care [5, 6]. For that reason, clinicians are encouraged to use guidelines to improve: 1) health outcomes in patients, 2) quality of clinical decisions by healthcare professionals, 3) efficiency of the healthcare system, 4) safety of care, and 5) cost effectiveness [11].

Although encouraged, guideline utilization by clinicians is suboptimal [12], even if barriers and facilitators of guideline utilization and adherence have been identified in the literature [11–13]. In their framework Cabana et al. identified nine barriers involved in physicians’ utilization: 1) lack of familiarity, 2) lack of awareness, 3) lack of agreement, 4) lack of self-efficacy, 5) lack of positive outcome expectancy, 6) lack of motivation, 7) external barriers, 8) patient-related barriers; and 9) context-related barriers. However, previous research focused on barriers related to one type of health care practitioners (mainly physicians) for one chronic disease such as diabetes, cancer, osteoarthritis, or low back pain [13].

Consequently, pathology and healthcare practitioners could be relevant factors (barriers or facilitators) of guidelines’ utilization. There is a lack of studies focusing on both barriers and facilitators of all practitioners involved in the management of one pathology. To our knowledge, no study has investigated the barriers and facilitators of guidelines’ utilization in all healthcare practitioners managing MSDs. This knowledge could inform implementation strategies. Therefore, we conducted a scoping review of the literature to describe the determinants of clinical practice guidelines’ utilization for the assessment and management of MSDs.

## Method

Our scoping review of the literature followed the methods proposed by Arksey and O’Malley [14–16] and included five steps: 1) identification of the research question; 2) identifying relevant studies; 3) selection of studies; 4) charting data with critical appraisal; and 5) collation, synthesis, and reporting results**.** Our review complies with the Reporting Items for Systematic Reviews and Meta-Analyzes extension for Scoping Reviews (PRISMA-ScR) statement [17]. We added one step to the methodology proposed by Arksey and O’Malley and critically appraised the methodological quality of relevant studies. Although quality assessment of studies is not yet a standard methodological step when conducting a scoping review, it is recommended in the PRISMA Scoping Review [17].

### Step 1: identifying the research question

Our research question was: “What are the determinants of use of clinical practice guidelines by healthcare providers for the assessment and management of musculoskeletal disorders?”

### Step 2: identifying relevant studies

A search strategy was developed in collaboration with a health-science librarian and reviewed by a second health-science librarian using the Peer Review of Electronic Search Strategies (PRESS) Checklist [18, 19] (See Appendix S1 for MEDLINE search strategy).

Three electronic databases (MEDLINE, Embase and AMED, through Ovid Technologies) were systematically searched from inception to March 12th, 2021. Search terms included subject headings specific to each database (e.g. MeSH in MEDLINE) and free text words relating to: “musculoskeletal disorders” AND “health practitioners” AND “guidelines”. The search strategy was first developed in MEDLINE and then adapted to the other bibliographic databases. We used the PRISMA-ScR flow chart to report number of articles at each stage [17].

### Step 3: study selection

#### Eligibility criteria

Eligible articles met the following inclusion criteria: 1) peer-reviewed articles published in English, French or Spanish; 2) investigation of determinants of guideline utilization focusing on barriers and facilitators (any factors that influence the utilization of evidence-based musculoskeletal guidelines); 3) source population included healthcare providers involved in the management of musculoskeletal disorders; and 4) epidemiological (controlled trials, cohort or cross-sectional studies), qualitative or mixed-method study designs. We excluded: 1) cadaveric or animal studies; and 2) books, book reviews, book chapters, conference abstracts, conference papers, editorials or letters to the editor, and literature reviews.

#### Screening

Two reviewers (DS and NL) independently screened all articles in two phases. In phase 1, reviewers screened titles and abstracts and classified articles as irrelevant, relevant, and possibly relevant. In phase 2, the full text of potentially relevant articles was reviewed for eligibility. When reviewers disagreed, they discussed until reaching consensus. If consensus could not be reached, the article was independently screened by a third reviewer (CD or PC) who discussed with the initial pair of reviewers to resolve disagreement.

### Step 4: charting data

#### Extraction of data

The lead author (DS) extracted data from all relevant studies and built an evidence Table. A second reviewer (NL) checked the validity of the data extraction. Extracted data included: 1) article identification (first author name, publication date and country); 2) study design (cross-sectional, cohort, randomized controlled trial; qualitative or mixed methods); 3) type of healthcare provider (DC: Doctor of Chiropractic; DO: Doctor of Osteopathy; MD: Medical Doctor; OT: Occupational Therapist; PT: Physiotherapist); 4) type of musculoskeletal disorders (low back pain, whiplash, neck pain, upper limbs, lower limbs, mixed MSDs); 5) guidelines related barriers and facilitators; and 6) quality of the study (low, medium, high).

#### Critical appraisal of studies

Pairs of reviewers (DS and NL) independently critically appraised relevant studies. Internal validity consensus of articles among reviewers was reached through discussion. A third independent reviewer was involved when consensus could not be reached (CD). For qualitative studies, methodological quality was assessed using the COnsolidated criteria for REporting Qualitative research Checklist (COREQ) [20]. For quantitative studies, methodological quality was assessed using the critical appraisal tools developed by Salmi [21]. The tool allows the classification of various study designs according to their methodological quality in one of 4 levels of internal validity: very good, quite good, low but acceptable, and unacceptable.

According to this classification, if the article complied with all internal-validity criteria in the quality checklist, the level of internal validity was assessed as “very good”. If the article missed some criteria but followed all major ones defined by the checklist, the level of internal validity was assessed as “quite good”. If one or more of those major criteria were missing, the level of internal validity was noted as “low but acceptable”. And, finally, if the article followed none of the criteria, the level of internal validity was “unacceptable”.

We did not exclude articles based with unacceptable quality. Rather, we used internal validity as a criteria to stratify the synthesis and classified studies in 3 categories: 1) low internal validity (gathering low but acceptable and unacceptable levels of the checklist) a; 2) moderate internal validity (quite good internal validity); and 3) high internal validity (very good level).

### Step 5: collation, synthesis, and reporting the results

We extracted the following data and synthesized them according to the two following steps:

1. Study characteristics: first author, year of publication, country, study design, healthcare provider(s), musculoskeletal disorder(s), methodological quality.
  Reported determinants of guidelines’ use: barriers and facilitators were extracted and classified according to the theory of planned behavior (TPB) [22]. This theory is the most frequent used classification to understand how determinants could influence clinicians’ practice [23]. This model approaches behavior by referring to three main concepts. The first concerns the attitudes and behavioral intention of healthcare providers toward guideline utilization. The second deals with the influence of subjective and social norms on guideline use. Finally, the third concept relates to perceived power and the perceived behavioral control.
  *Attitudes* toward guideline use focusses on clinicians’ cognitive and emotional beliefs about their behavior, and individual positive or negative evaluation of the use of guidelines. This part includes the intention to perform a given behavior and therefore refers to motivational factors that may influence a behavior: if you have a strong intention to perform the behavior, you will perform it.
  *The subjective norms* refer to the perception of usual behavior by other peoples, and the perceived pressure to comply with this behavior, including an individual’s motivation and cultural or social context influence.
  *The perceived behavioral control* deals with the availability of skills needed to carry out the behavior, including expected external factors such as available resources and opportunities (e.g. skills, time, and cooperation of others). This part deals with factors linked with perceived ease or difficulty to perform a behavior.
  These three concepts can influence each other. The more favorable the attitude and subjective norm with respect to a given behavior and the greater the perceived control, the stronger the intention to perform the behavior. Thus, barriers can be obstacles to the intention to perform the expected behavior—in our case guidelines utilization—and thus act as negative predictors of intention, because they contribute to an unfavorable evaluation of the use of guidelines. Obviously, facilitators act as positive predictors of intention and thus contribute to a favorable evaluation of the use of guidelines.
2. Interpretation of findings: We used mind mapping [24] to synthesize barriers or facilitators as a visual interpretation.

## Results

**Fig. 1:**
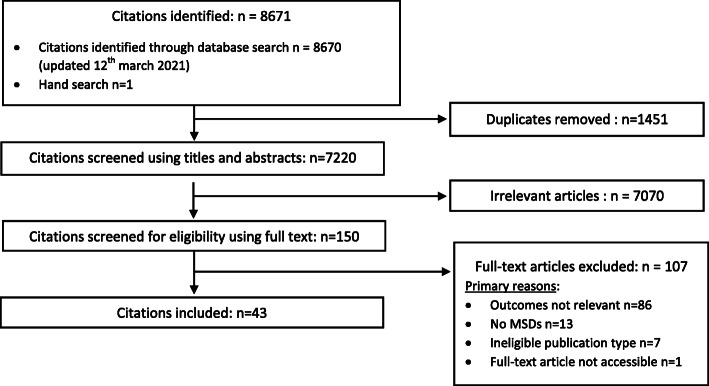
Flow chart of articles screening

Our search retrieved 8671 citations (Fig. 1). After removing duplicates (*n* = 1451), we screened 7220 articles. During the phase 1 screening, 7070 articles were irrelevant, and 150 full text were examined. During the phase 2 screening, 107 articles were excluded for the following reasons: 1) outcomes did not focus on guideline utilization (*n* = 86); 2) MSDs were not the only condition studied and the results were not stratified by disorders (*n* = 13); 3) publication type was not eligible (commentaries et poster presentation) (*n* = 7); 4) full-text article was not available (n = 1). Therefore, our synthesis included 43 articles (Table 1) [4, 25–60].

**Table 1:** Description of articles included classified by year of publication (*n* = 43)

First author Year of publication	Country	Design	Healthcare providers	Musculoskeletal disorder (MSDs)	Quality of the study
Biggs et al. 1997 [25]	North America	Quantitative (cross-sectional)	DC (*n* = 401)	Mixed MSDs	Low
Schers et al. 2000 [26]	Europe	Quantitative (cohort)	MD (*n* = 30)	Low back pain	High
Hurley et al. 2002 [27]	North America	Quantitative (cross-sectional)	PT (*n* = 118)	Neck pain	Low
Espeland et al. 2003 [28]	Europe	Qualitative	MD (*n* = 13)	Low back pain	Low
Schectman et al. 2003 [29]	North America	Quantitative (RCT)	MD (*n* = 53)	Low back pain	Low
Baker et al. 2006 [30]	Europe	Qualitative	MD (*n* = 29)	Low back pain	Low
Coudeyre et al. 2006 [31]	Europe	Quantitative (cross-sectional)	MD (*n* = 864)	Low back pain	Low
Dahan et al. 2006 [32]	Israel	Qualitative	MD (*n* = 38)	Low back pain	Low
Poiraudeau et al. 2006 [33]	Europe	Quantitative (cross-sectional)	MD (*n* = 266)	Low back pain	Medium
Bishop et al. 2008 [34]	Europe	Quantitative (cross-sectional)	Mixed providers (*n* = 1022)	Low back pain	Medium
Chenot et al. 2008 [35]	Europe	Quantitative (cross-sectional)	MD (*n* = 72)	Low back pain	Low
Corbett et al. 2009 [36]	Europe	Mixed method	MD (*n* = 16)	Low back pain	Medium
Côté et al. 2009 [37]	North America	Qualitative	PT (*n* = 16)	Low back pain	High
Harting et al. 2009 [38]	Europe	Qualitative	PT (*n* = 30)	Low back pain	High
Rutten et al. 2009 [39]	Europe	Quantitative (cross-sectional)	PT (*n* = 232)	Low back pain	Medium
Fullen et al. 2011 [40]	Europe	Quantitative (cross-sectional)	MD (*n* = 432)	Low back pain	High
Kooijman et al. 2011 [41]	Europe	Quantitative (cross-sectional)	PT (*n* = 121)	Lower limb	Low
Poitras et al. 2011 [42]	North America	Qualitative	OT (*n* = 9)	Low back pain	High
Bussières et al. 2012 [4]	North America	Qualitative	DC (*n* = 21)	Low back pain	High
Jeffrey et al. 2012 [43]	North America	Qualitative	PT (*n* = 11)	Low back pain	Low
Pincus et al. 2012 [44]	Europe	Quantitative (cross-sectional)	Mixed providers (*n* = 465)	Low back pain	Medium
Poitras et al. 2012 [45]	North America	Qualitative	Mixed providers (*n* = 32)	Low back pain	High
Simmonds et al. 2012 [46]	North America	Quantitative (cross-sectional)	PT (*n* = 100)	Low back pain	Medium
Matzon et al. 2013 [47]	Europe	Quantitative (cross-sectional)	PT (*n* = 187)	Low back pain	Low
Hendrick et al. 2013 [48]	NZ-Australia	Quantitative (cross-sectional)	PT (*n* = 170)	Low back pain	Low
Rebbeck et al. 2013 [49]	NZ-Australia	Quantitative (cohort)	Mixed providers (*n* = 80)	Whiplash	High
Corkery et al. 2014 [50]	North America	Quantitative (cross-sectional)	PT (*n* = 291)	Whiplash	Low
Gremeaux et al. 2014 [51]	Europe	Quantitative (cross-sectional)	MD (*n* = 47)	Low back pain	Medium
Learman et al. 2014 [52]	North America	Quantitative (cross-sectional)	PT (*n* = 1144)	Low back pain	Medium
Bishop et al. 2015 [53]	Europe	Qualitative	Mixed providers (*n* = 53)	Low back pain	Medium
Bernhardsson et al. 2015 [54]	Europe	Quantitative (cross-sectional)	PT (*n* = 271)	Mixed MSDs	Low
Clement et al. 2015 [55]	North America	Quantitative (cross-sectional)	Mixed providers (*n* = 456)	Neck pain	Low
Maas et al. 2015 [56]	Europe	Mixed method	PT (*n* = 44)	Low back pain	Low
Ladeira et al. 2015 [57]	North America	Quantitative (cross-sectional)	PT (*n* = 327)	Low back pain	Medium
Brijnath et al. 2016 [58]	North America	Quantitative (cross-sectional)	MD (*n* = 423)	Whiplash	Medium
Derghazarian et al. 2016 [59]	North America	Quantitative (cross-sectional)	PT (*n* = 108)	Low back pain	High
Epstein-Sher et al. 2017 [60]	Israel	Quantitative (cross-sectional)	MD (*n* = 86)	Low back pain	Low
Figg-Latham et al. 2017 [61]	Europe	Qualitative	DO (*n* = 12)	Low back pain	High
Suman et al. 2017 [62]	Europe	Mixed method	Mixed providers (*n* = 96)	Low back pain	Low
Stilwell et al. 2017 [63]	North America	Qualitative	Dc (*n* = 13)	Low back pain	High
Cowell et al. 2018 [64]	Europe	Qualitative	PT (*n* = 10)	Low back pain	Medium
Selby et al. 2018 [65]	Europe	Quantitative (cross-sectional)	GP (*n* = 167)	Low back pain	High
Akindele et al. 2019 [66]	Africa	Quantitative (cross-sectional)	PT (*n* = 189)	Low back pain	High

*DC* Doctor of Chiropractic, *DO* Doctor of Osteopathy, *MD* Medical Doctor, *MSDs* Musculoskeletal Disorder, *NZ* New Zealand, *OT* Occupational Therapist, *PT*, Physiotherapist, *RCT*, Randomized Clinical Trial

### Study characteristics

Most articles (67%, 29/43) were published after 2009, 30% (*n* = 12) between 2001 and 2009, and 3% (*n* = 2) were published before 2001 (Table 1). Studies were mainly conducted in Europe (51%, *n* = 22), and North America (35%, *n* = 15), with a lower proportion elsewhere (7% in New Zealand or Australia (*n* = 3), 5% in Israel (n = 2) and 2% in Africa (n = 1)). Low back pain was the most studied disorder (81%, *n* = 35), followed by neck pain and associated disorders (12%, *n* = 5), lower limb disorders (*n* = 1), and two articles evaluated mixed MSDs. Most studies (63%, *n* = 27) used epidemiological designs, 30% (*n* = 13) used qualitative designs and 7% (n = 3) used mixed methods. Most epidemiological studies were cross-sectional (85%, *n* = 23), 11% (n = 3) were cohorts, and one was a randomized controlled trial. Forty percent of studies (*n* = 17) investigated physiotherapists; 30% (n = 13) medical doctors; 9% (*n* = 4) doctors of chiropractic; 2% (n = 1) occupational therapists; 2% (n = 1) doctors of osteopathy; and 16% (*n* = 7) investigated multiple professions.

Regarding methodological quality, 42% (*n* = 18), 30% (*n* = 12) and 30%(n = 13) were of low, medium and high-quality level, respectively.

Finally, we found no difference between determinants related to internal validity, country, type of healthcare providers or MSDs.

### Barriers to guidelines’ utilization

**Fig. 2:**
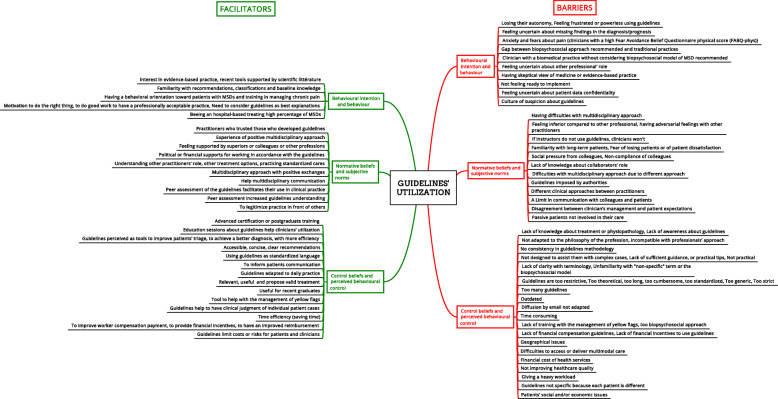
Determinants of guidelines ‘utilization emerging from the literature

Barriers to guidelines’ utilization are reported in Fig. 2 according to their frequency of citation in the literature and detailed below according to the planned behavior theory.

#### Barriers linked to clinicians’ attitudes towards their behavior

Clinicians who feel frustrated, anxious or a perceived loss of autonomy when using guidelines were less likely to use them [4, 27, 28, 32, 38, 43, 63, 64, 67]. Furthermore, being afraid of missing information such as clinical signs or patient information, was also perceived as a barrier to use guidelines [4, 26, 28, 32]. The perception of a gap between the biopsychosocial model of care recommended by guidelines and their current practice (for example biomedical approach) [28, 31, 33, 51, 63, 64], a culture of suspicion about guidelines [36], and a skeptical view of medicine or evidence-based practice [53, 61] were identified as barriers of guideline utilization.

#### Barriers included in subjective norms

We identified barriers related to clinicians’ judgment and perception of their own behavior by other people. Health care providers reported to be influenced by non-compliance of their instructors during their training [4, 61], non-compliance of colleagues or other professionals in their practice [4, 28, 30, 32, 36, 45, 55, 67]. For example, when they had experienced the feeling of being in competition with other practitioners [36, 61]. Moreover, authorities and public health policies could have an impact on clinicians perceiving guidelines as mandatory [36]. Finally, long-term patients could also have an impact on guidelines’ utilization; clinicians could be afraid to lose these patients if they do not satisfy their expectations [30, 35, 61, 63–65].

#### Barriers involved in clinicians perceived behavioral control

Barriers were related to clinicians’ perception of how they control their behavior. Guidelines may be perceived as non-practical for current practice [26, 28, 29, 32, 41, 45, 58, 62, 64–66] or reported to be too restrictive, theoretical, long, cumbersome [4, 27, 37, 38, 53, 56, 63, 64, 66, 67] or outdated [61, 67]. The number of available guidelines may be viewed as too large for practitioners [36, 38], with a lack of consistency in their methodology [4, 28, 38, 42, 45, 66, 67]. Consequently, clinicians may be confused when selecting a guideline. Furthermore, terminology used is sometimes perceived to be unclear, particularly regarding the term “non-specific” used to describe some MSDs such as neck or low back pain [32, 37, 38, 43, 45, 53, 60, 62].

In addition, some clinicians reported that they are not sufficiently trained to use guideline recommendations. For example, clinicians who are not trained to use yellow flags, or the biopsychosocial approach would be challenged with using them to manage patients [30, 34, 37, 42, 45, 46, 48, 59, 63, 64, 67]. Barriers to compliance also include the ability to provide recommended multimodal care, and accessibility and reimbursement for healthcare services [30, 32, 35, 42, 45, 53, 58, 66, 67]. For these reasons, some practitioners perceived guidelines as not adapted to the needs of their patients, limiting the interest in using guidelines to inform care for individual cases.

### Facilitators to guidelines’ utilization

All facilitators to guideline utilization are reported in Fig. 2.

#### Facilitators linked to clinicians’ attitudes towards their behavior

A practitioner’s motivation to provide good clinical care, positive behaviors toward using guidelines and professionalism were frequently associated with compliance [4, 28, 35]. Providers interested in scientific literature used guidelines more frequently [4, 32, 36, 42, 50, 53, 67]. Clinicians who are interested in evidence-based practice are more likely to use recommendations from guidelines [49, 50, 60]. Furthermore, clinicians practicing in a hospital or a clinic with a large volume of MSDs patients reported using guidelines more often [41, 52, 57].

#### Facilitators included in subjective norms

These determinants involved how clinicians practice and interact with others [62]. Relationships and social interactions with colleagues, superiors, and public health authorities can influence guideline utilization [39, 45, 55, 56, 63, 66]. Moreover, clinicians who use recommendations prefer to perceive that the guidelines are commonly used in practice by others in their field [37, 42]. Having good experiences with recommended multidisciplinary approaches [62] encourages clinicians to maintain this behavior in practice. In this way, they use guidelines as a common and shared language between different professions [38, 39, 42, 61]. Clinicians who want to legitimize their own practice in front of others [61] use guidelines in practice. Finally, it is reported that clinicians need to trust those who developed guidelines [4, 38] and must have financial resources supported by authorities to work in accordance with guidelines [38, 39, 63].

#### Facilitators involved in clinicians’ perceived behavioral control

Determinants may be linked to clinicians’ perception of control about their ability to use guidelines. Recommendations must be perceived as accessible, concise, clear, adapted to daily practice, useful and relevant for use by clinicians [32, 37, 39, 42, 63, 67].

Providers who aim to improve their practice tend to use more guidelines. Practice improvement occurs when providers view guidelines s tools to help form clinical judgments, inform patient communication, improve patients’ triage, and be more efficient [35, 39, 45, 53, 55, 58, 63]. Some clinicians expressed the need to be trained by having access to education sessions about guidelines utilization [29, 66].

## Discussion

### Summary of evidence

Our scoping review provides an overview of determinants related to guideline utilization by health care providers. Most studies focusing on guideline utilization used a cross-sectional design. Barriers to guideline utilization were more common than facilitators. We did not find any difference between determinants influencing utilization of guidelines related to healthcare provider or MSDs. This suggests that barriers and facilitators highlighted in this scoping review may apply to various healthcare providers who manage MSDs. The majority of determinants identified in this review were reported from high or moderate internal validity studies.

We note that only a few determinants associated with patient characteristics have been identified, underscoring the fact that the literature on guideline use is largely focused on clinician characteristics.

Our results suggest that obstacles and facilitators tend to hold opposite views. For example, guideline users perceive them as adaptable to daily practice because they are relevant, useful, accessible, concise and clear. Alternatively, non-users perceive guidelines as not improving healthcare quality because they are restrictive, cumbersome, theoretical, too plentiful, and time consuming.

According to clinicians’ behavior, two concepts that influence guideline utilization depend on a clinician’s perception of the factor (Fig. 2). The first concept is a positive practice orientation such as feeling comfortable with the management of yellow flags and biopsychosocial factors which are supported by guidelines. On the other hand, clinicians with a biomedical practice seem to have difficulties considering the biopsychosocial model recommended by guidelines. Second, the habit of working with this kind of tool is important. We can differentiate between clinicians who are familiar with recommendations or classifications and those who are not, and consequently feel frustrated or powerless using these tools.

We found that perception of clinical behavior by colleagues, other professionals or authorities could have both negative and positive influence on clinicians’ guideline utilization. Moreover, past experiences can enhance or limit the use of guidelines. Having a positive experience with others when guidelines recommend multidisciplinary approaches in the management of a patient can encourage practitioners to repeat the experience and apply guidelines. On the contrary, when clinicians feel at a disadvantage compared to other professions, or experience disagreement without explanations about multidisciplinary management of a patient, they may not use guidelines. Finally, authorities impact the use of guidelines through the diffusion of tools and by adding value to clinical management following guideline recommendations. Clinicians use guidelines if they feel supported to work in accordance with recommendations. If they feel they are not supported and perceive guidelines as imposed, their attitude toward the use of guidelines is negative.

### Comparison with previous reviews of the literature

Our results concerning barriers to guideline utilization agree with previous studies [13, 68]. Cabana et al. also reported that barriers to guideline use include: lack of awareness, lack of familiarity, lack of agreement with specific guidelines or guidelines in general, lack of self-efficacy, lack of outcome expectancy, lack of motivation, external barriers linked with patient factors, guideline factors or environmental factors [13]. Furthermore, in their scoping review, Fischer et al. identified the following barriers among physicians: lack of awareness, a lack of familiarity, lack of agreement, self-efficacy, skills, outcome expectancy and motivation [68]. However, our scoping review adds to the literature by identifying a complete list of barriers and facilitators of guideline utilization in a broad range of health care providers who manage patients with MSDs. Importantly, our review found no association between determinants and professions. Our determinants are classified using a standardized framework and visually displayed in a mind-map (Fig. 2).

The evidence included in our scoping review suggest that the recommendations focus on altering the behaviors of guidelines consumers. However, one possible barrier which was not discussed in the literature is that the guidelines themselves are often inconsistent in their methodology and recommendations. Therefore, we recommend that the AGREE reporting checklist and RIGHT statement be used for the reporting of clinical practice guidelines [69, 70].

### Strengths and limitations

A strength of our scoping review is its comprehensive scope. We focused our research on all MSDs to emphasize the importance of patient-centered care for these disorders in a multidisciplinary approach. This clinical situation involves all healthcare providers and data were described from a clinical point of view.

We used the Theory of Planned Behavior. This is the most frequently classification used in implementation science [13]. This theory helps identify healthcare providers’ determinants of intension to use, their use of guidelines, and their beliefs towards guidelines. This is a major step in developing targeted interventions for clinicians’ adherence. Future interventions must focus on maintaining, sustaining and encouraging guideline use, and thus encourage positive behaviors that improve diagnosis and patient management. Finally, we performed a critical appraisal, a limitation mentioned in previous scoping reviews on this topic [17]. Our critical appraisal revealed that two thirds of determinants were extracted from high or medium quality studies. One third of determinants, therefore, needs to be carefully considered. Our scoping review has some limitations. Because we searched only three databases (adhering to standard scoping review methodology), we may have missed some studies. Also, we did not search the grey literature, and we restricted languages to English, Spanish or French.

## Conclusion and perspectives

Implementation strategies need to be oriented toward determinants expressed by clinicians, the major users of guidelines. Implementation tools must be developed that are tailored to clinicians’ expectations and that are informed by facilitators of utilization and that avoid barriers to orient work on implementation strategies. Our study identifies determinants for public health policy makers and professional associations that need to be prioritized when implementing guidelines. Whether the use of guidelines varies according to the social characteristics of patients could also be an interesting question for further research.

## Supplementary Information

**Additional file 1: Appendix 1**. Search strategy in Medline.

## Data Availability

Raw data are available on request to the first author dsorondo@ifec.net
