# Supplementary material for: Determinants of clinical practice guidelines’ utilization for the management of musculoskeletal disorders: a scoping review
Source: BMC Musculoskelet Disord. 2021 Jun 1;22:507. doi: 10.1186/s12891-021-04204-w (PMC8170973; doi:10.1186/s12891-021-04204-w)
Supplement: Supplementary file 1 — Additional file 1: Appendix 1. Search strategy in Medline. [file 12891_2021_4204_MOESM1_ESM.pdf]

Appendix 1 Search strategy in Medline

Search run March 10, 2018 in Ovid MEDLINE: Epub Ahead of Print, In-Process & Other Non-Indexed Citations, Ovid MEDLINE® Daily and Ovid MEDLINE® 1946-Present: 2871 results. The search was updated on December 1st, 2019 and on March 12th, 2021.

#	Searches
1	Whiplash Injuries/
2	Headache/
3	Post-Traumatic Headache/
4	Tension-Type Headache/
5	Neck Injuries/
6	Neck Pain/
7	Neck Muscles/in [Injuries]
8	exp Cervical Vertebrae/in [Injuries]
9	Radiculopathy/
10	exp Brachial Plexus Neuropathies/
11	exp Torticollis/
12	whiplash.ab,kw,ti.
13	"neck injur* ".ab,kw,ti.
14	"neck pain* ".ab,kw,ti.
15	"neck ache* ".ab,kw,ti.
16	"neckache* ".ab,kw,ti.
17	(headache* adj3 (cervicogenic or tension)).ab,kw,ti.
18	"brachial plexus neuropath* ".ab,kw,ti.
19	torticollis.ab,kw,ti.
20	or/1-19 [**neck injuries]
21	exp Back Injuries/
22	exp Back Pain/
23	Coccyx/in [Injuries]
24	Intervertebral Disc Degeneration/
25	Intervertebral Disc Displacement/
26	Lumbar Vertebrae/in [Injuries]
27	Lumbosacral Region/in [Injuries]
28	Osteoarthritis, Spine/
29	Piriformis Muscle Syndrome/
30	Sciatica/
31	Spinal Diseases/
32	Spinal Stenosis/
33	(back adj3 (ache* or disorder* or dysfunction or injur* or pain*)).ab,kw,ti.
34	(backache* adj3 (injur* or pain*)).ab,kw,ti.
35	(back pain or back-pain).ab,kw,ti.
36	(lumbar disc* adj3 (extruded or degenerat* or herniat* or prolapse* or sequestered or slipped)).ab,kw,ti.
37	(lumbar disk* adj3 (extruded or degenerat* or herniat* or prolapse* or sequestered or slipped)).ab,kw,ti.
38	"low* back pain ".ab,kw,ti.
39	(lumbar adj3 (pain or facet or nerve root* or osteoarthritis or radicul* or spinal stenosis or spondylo* or zygapophys*)).ab,kw,ti.
40	"Piriformis syndrome* ".ab,kw,ti.
41	(sacral adj2 pain*).ab,kw,ti.

42	sciatica.ab,kw,ti.
43	((spine or spinal) adj3 (condition* or disable* or disabilit* or disorder* or pain or stenos?s)).ab,kw,ti.
44	spondylosis.ab,kw,ti.
45	or/21-44 [**back injuries]
46	Shoulder Pain/
47	exp Cumulative Trauma Disorders/
48	exp Median Neuropathy/
49	Shoulder Impingement Syndrome/
50	Shoulder Joint/in [Injuries]
51	Shoulder/in [Injuries]
52	exp Arm Injuries/
53	exp Hand Injuries/
54	Wrist Injuries/
55	Finger Injuries/
56	exp Tendinopathy/
57	Radial Neuropathy/
58	exp Ulnar Neuropathies/
59	Thoracic Outlet Syndrome/
60	"carpal tunnel syndrome* ".ab,kw,ti.
61	(medial adj3 (epicondylitis or epicondylosis or epicondylopathy)).ab,kw,ti.
62	(lateral adj3 (epicondylitis or epicondylosis or epicondylopathy)).ab,kw,ti.
63	(shoulder* adj3 (pain* or sprain* or strain* or injur* or impair* or impingement)).ab,kw,ti.
64	(shoulder* adj3 (tendinopathy or tendinitis or tendonitis or capsulitis)).ab,kw,ti.
65	((glenohumeral or scapul* or acromioclavicular) adj3 (pain* or sprain* or strain* or injur*)).ab,kw,ti.
66	(rotator cuff adj3 (sprain* or strain* or tear* or bursitis tendinitis or impingement)).ab,kw,ti.
67	((supraspinatus or infraspinatus or subscapularis or teres minor or teres major or trapezius or deltoid or bicep* or bicipital or coracobrachialis) adj3 (impingement or strain* or tear* or pain*)).ab,kw,ti.
68	biceps tend?nitis.ab,kw,ti.
69	painful arc.ab,kw,ti.
70	frozen shoulder.ab,kw,ti.
71	((shoulder and capsul*) adj3 (sprain* or tear*)).ab,kw,ti.
72	(arm* adj3 (pain* or sprain* or strain* or injur* or impair*)).ab,kw,ti.
73	(wrist* adj3 (pain* or sprain* or strain* or injur* or impair*)).ab,kw,ti.
74	(hand* adj3 (pain* or sprain* or strain* or injur* or impair*)).ab,kw,ti.
75	(elbow* adj3 (pain* or sprain* or strain* or injur* or impair*)).ab,kw,ti.
76	"thoracic outlet syndrome* ".ab,kw,ti.
77	tennis elbow.ab,kw,ti.
78	(rotator cuff adj3 (injur* or disorder*)).ab,kw,ti.
79	(median adj neuropath*).ab,kw,ti.
80	(radial adj neuropath*).ab,kw,ti.
81	"upper extremit* injur* ".ab,kw,ti.
82	((radial or ulnar) adj neuropath*).ab,kw,ti.
83	"cumulative trauma disorder* ".ab,kw,ti.
84	(repetit* adj3 (strain* or sprain* or injur* or disorder*)).ab,kw,ti.
85	or/46-84 [**upper extremity injuries]
86	exp Hip Injuries/
87	exp Leg Injuries/
88	exp Knee Injuries/
89	exp Foot Injuries/
90	exp Toes/in [Injuries]
91	Ankle Injuries/

92	Lateral Ligament, Ankle/in [Injuries]
93	Fasciitis, Plantar/
94	(lower adj3 (extremit* or limb* or injur*)).ab,kw,ti.
95	(ankle* adj3 (sprain* or strain* or injur*)).ab,kw,ti.
96	((talofibular or calcaneofibular or calcaneotibial or tibio*) adj3 (sprain* or strain* or injur*)).ab,kw,ti.
97	(buttock* adj3 (injur* or pain*)).ab,kw,ti.
98	(foot adj3 (injur* or pain*)).ab,kw,ti.
99	(hip* adj3 (injur* or pain*)).ab,kw,ti.
100	(knee* adj3 (injur* or pain*)).ab,kw,ti.
101	(leg* adj3 (injur* or pain*)).ab,kw,ti.
102	(thigh* adj3 (injur* or pain*)).ab,kw,ti.
103	(toe* adj3 (injur* or pain* or turf)).ab,kw,ti.
104	"patellofemoral pain syndrome* ".ab,kw,ti.
105	tendinosis.ab,kw,ti.
106	tendinopathy.ab,kw,ti.
107	plantar fasciitis.ab,kw,ti.
108	or/86-107 [**lower extremity injuries]
109	Musculoskeletal Diseases/
110	((musculoskeletal or musculo-skeletal or MSK) adj4 (care or condition* or disabilit* or disorder* or injur* or pain or problem* or trouble*)).ab,kw,ti.
111	Arthritis/
112	arthritis.ab,kw,ti.
113	Bursitis/
114	bursitis.ab,kw,ti.
115	exp Osteoarthritis/
116	osteoarthritis.ab,kw,ti.
117	Spondylitis, Ankylosing/
118	(ankylosing adj2 spondylitis).ab,kw,ti.
119	exp Temporomandibular Joint Disorders/
120	temporomandibular.ab,kw,ti.
121	or/109-120 [**MSK general]
122	20 or 45 or 85 or 108 or 121 [**MSK disorders]
123	Acupuncture/
124	Acupuncture Therapy/
125	Allied Health Personnel/
126	Caregivers/
127	Case Managers/
128	Chiropractic/
129	Dentists/
130	Faculty, Medical/
131	Faculty, Nursing/
132	General Practitioners/
133	Health Educators/
134	Hospitalists/
135	Manipulation, Chiropractic/
136	exp Medical Staff/
137	Medicine, Chinese Traditional/
138	Neurologists/
139	Neurosurgeons/
140	exp Nurses/
141	Nurses' Aides/

142	exp Nursing/
143	Occupational Health Physicians/
144	Occupational Therapists/
145	Occupational Therapy/
146	Orthopedic Surgeons/
147	exp ORTHOPEDICS/ma [Manpower]
148	Osteopathic Physicians/
149	Physiatrists/
150	Physical Therapists/
151	Physical Therapist Assistants/
152	Physical Therapy Modalities/
153	Physical Therapy Specialty/
154	Physicians/
155	exp Physician Assistants/
156	Physician-Patient Relations/
157	Physicians, Family/
158	Physicians, Primary Care/
159	Physicians, Women/
160	Practice Patterns, Physicians'/
161	Primary Health Care/
162	Podiatry/
163	Professional-Patient Relations/
164	exp Radiologists/
165	Rheumatologists/
166	"acupuncturist*".ab,kw,ti.
167	(chiropractic adj2 (physician* or doctor* or practitioner*)).ab,kw,ti.
168	"chiropractor*".ab,kw,ti.
169	dentist.mp. or dentists.ab,kw,ti. [mp=title, abstract, original title, name of substance word, subject heading word, keyword heading word, protocol supplementary concept word, rare disease supplementary concept word, unique identifier, synonyms]
170	(faculty adj3 (medical or nursing)).ab,kw,ti.
171	"general adj2 practitioner*".ab,kw,ti.
172	"hospitalist*".ab,kw,ti.
173	"manual adj2 therapist*".ab,kw,ti.
174	naprapath*.ab,kw,ti.
175	"neurologist*".ab,kw,ti.
176	"nurse*".ab,kw,ti.
177	(occupational adj2 (physician* or therapist*)).ab,kw,ti.
178	((orthopedic or orthopaedic) adj2 surgeon*).ab,kw,ti.
179	"osteopath*".ab,kw,ti.
180	"physiatrist*".ab,kw,ti.
181	(physical adj therapist*).ab,kw,ti.
182	"physiotherapist*".ab,kw,ti.
183	"physician*".ab,kw,ti.
184	(primary adj care).ab,kw,ti.
185	"podiatrist*".ab,kw,ti.
186	(psychomotor or psycho-motor or psychomotricien).ab,kw,ti.
187	"radiologist*".ab,kw,ti.
188	"rheumatologist*".ab,kw,ti.
189	(spine adj3 specialist*).ab,kw,ti.
190	traditional Chinese medicine.ab,kw,ti.

191	or/123-190 [**health practitioners]
192	Guidelines as Topic/
193	Practice Guidelines as Topic/
194	Guideline Adherence/
195	Health Planning Guidelines/
196	Clinical Protocols/
197	Consensus Development Conferences as Topic/
198	guideline.pt.
199	practice guideline.pt.
200	consensus development conference.pt.
201	(guideline* adj3 (clinical or consensus or practice or development of validate* or standard*)).ab,kw,ti.
202	(guideline* adj3 (accept* or adher* or adopt* or attitude* or barrier* or belief* or benchmark* or bench mark* or compliance* or comply* or determinant* or diffusion or disseminat* or distribut* or effective* or enable*)).ab,kw,ti.
203	(guideline* adj3 (impact* or implement* or introduc* or knowledge or motivat* or transfer* or translat* or uptake or use* or utilisat* or utilizat*)).ab,kw,ti.
204	(guideline* adj3 (impact* or implement* or introduc* or knowledge or motivat* or transfer* or translat* or uptake or use* or utilisat* or utilizat*)).ab,kw,ti.
205	(consensus adj3 (conference* or process* or statement*)).ab,kw,ti.
206	(evidence-based adj3 (approach* or guideline* or recommendation* or consensus or position statement*)).ab,kw,ti.
207	(evidence based adj3 (approach* or guideline* or recommendation* or consensus or position statement*)).ab,kw,ti.
208	(evidence-informed adj3 (approach* or guideline* or recommendation* or consensus or position statement*)).ab,kw,ti.
209	(evidence informed adj3 (approach* or guideline* or recommendation* or consensus or position statement*)).ab,kw,ti.
210	(practice adj2 parameter*).ab,kw,ti.
211	or/192-210 [**guidelines]
212	122 and 191 and 211
213	limit 212 to (english or french or spanish)
